# Recycling and Degradation of Polymeric Materials: Exploring Different Perspectives in Plastic Waste Management

**DOI:** 10.3390/ma19040682

**Published:** 2026-02-11

**Authors:** Giovanni Gadaleta, Andrea Sorrentino, Maria Oliviero, Sabino De Gisi

**Affiliations:** 1 Biodegradability and Compostability Laboratory, AIMPLAS–Plastics Technology Centre, C. Gustave Eiffel n. 4, 46980 Paterna, Spain; 2 Institute of Polymers, Composites and Biomaterials (IPCB), National Research Council (CNR), P.le E. Fermi 1, 80055 Portici, Italy; 3 Department of Industrial Engineering, Section of Chemical Engineering, University of Salerno, Via Giovanni Paolo II n. 132, 84084 Fisciano, Italy

Plastics are indispensable in modern societies because they offer a broad portfolio of properties at comparatively low cost, enabling applications ranging from packaging and consumer goods to transportation, electronics, construction, and healthcare [1]. However, the same attributes that make polymers attractive (such as durability, chemical resistance, and design flexibility) also contribute to persistent environmental burdens when products are mismanaged [2]. Over recent decades, the combined effects of rapidly increasing production, short product lifetimes in key markets (packaging for instance), and inadequate collection and treatment infrastructures have resulted in large and growing waste streams as a result of either improper recycling or leakage into natural environments [3]. This context highlights that improvements across the entire life cycle of plastic are necessary (from design and material choice to collection, sorting, recycling, and end-of-life management) to overcome the current situation and focus on environmental protection and climate goals.

“Recycling” cannot be treated as a single, uniform solution. Mechanical recycling, when well organized and of a high quality, is central to reducing resource demand and emissions in thermoplastic production. However, it is frequently constrained by contamination, polymer–polymer incompatibilities, additive legacies, and property deterioration associated with thermo-mechanical and thermo-oxidative degradation [4,5,6]. Chemical recycling routes (covering depolymerization, solvolysis, and other conversion strategies) have been investigated as complementary options, particularly for complex, multi-material, or highly degraded streams, as well as for polymer classes that are intrinsically difficult to reprocess mechanically [7,8]. In parallel, the expansion of biodegradable bioplastics has opened another “biological” treatment of some products, especially in products contaminated by food waste or difficult to sort from organic matter (e.g., mulching film) [9].

This Special Issue was launched to address a gap that is often visible in literature: recycling performance is frequently discussed without fully integrating the chemistry and physics of degradation and degradation is sometimes studied in isolation from the technological and regulatory realities of end-of-life management. The six contributions assembled in the Special Issue collectively examine polymer waste management through complementary lenses: (i) the chemical recycling of rigid polyurethane foams [10], (ii) degradation control and additive dynamics in polypropylene mechanical recycling under open- and closed-loop conditions [11], (iii) the regulatory-oriented assessment of plastics recovered from waste mobile phones under EU chemical and product restrictions [12], (iv) the anaerobic biodegradation of PLA-based disposable items under mesophilic digestion conditions [13], (v) a comprehensive review on bioplastic biodegradability across managed and unmanaged environments [14], and (vi) the thermal and chemical characterization of reprocessed PET from commercial and recycled sources, including bottle-grade and textile-related blends [15].

A key issue with plastics is the management of rigid polyurethane (PU) foams, which are widely used for insulation and other high-value applications. They are challenging to recycle mechanically due to their crosslinked structure and the diversity of formulations used to provide the necessary properties [16]. As a result, chemical recycling pathways such as glycolysis and related solvolysis approaches are frequently employed to recover polyol-rich fractions and to enable partial material circularity in PU value chains [17]. Zemła et al. [10] investigated how the chemical structure and apparent density of rigid PU foams influence the properties of chemical recycling products. By explicitly connecting foam structure and macroscopic foam characteristics to recycling outcomes, this study contributes to a more “structure–process–product” understanding of PU recycling. This approach is particularly important for thermosets and highly formulated polymers, where feedstock variability can dominate process performance.

The main limiting factor for polyolefins, which dominate packaging markets, is often not whether the material can be re-melted, but whether the resulting recyclate can meet functional and regulatory requirements after repeated processing [18]. Thermo-oxidative degradation in polypropylene (PP) is a central concern because it can drive chain scission and property drift, while additives introduced to stabilize materials can themselves create complications when they accumulate across cycles or generate transformation products [19]. Knoben et al. [11] addressed this challenge by focusing on how antioxidants behave when rigid PP is mechanically recycled, comparing two scenarios: open-loop recycling (the material leaves the original application) and closed-loop recycling (the material is recycled back into the same application). Their main methodological novelty is a modified differential scanning calorimetry approach to determine which oxidation induction temperature (OIT) is safer and more suitable for real recycled PP, where unknown contaminants can otherwise make standard OIT tests problematic. Using this approach, they show that OIT can be used as a practical indicator of the remaining “active” antioxidant level and, therefore, of the thermo-oxidative stability of the recycled PP. The study also illustrates an important practical trade-off: if too little antioxidant remains, stability drops and further reprocessing becomes risky; however, if antioxidants are repeatedly re-added in closed-loop schemes, they may accumulate over cycles, which makes additive management a key aspect of high-quality recycling.

Poly(ethylene terephthalate) (PET) provides a different but equally instructive case. PET has one of the most established recycling value chains, including bottle-to-bottle routes in many jurisdictions, yet reprocessing still alters molecular and chemical features in ways that can influence processability, odor, color, and long-term performance [20]. These effects become more complex when recycled PET is blended across sources and applications (e.g., bottle-grade streams mixed with textile-derived fractions), which can introduce variability in additives, copolymers, and degradation history [21]. Gomes et al. [15] contributed to this discussion through a thermal and chemical characterization study comparing commercial, recycled, bottle-grade, and textile blend PET materials. Recyclers in PET markets must often manage heterogeneous inputs and still deliver predictable material specifications. This work aligns closely with these operational realities by emphasizing comparative characterization rather than idealized single-stream assumptions.

Electronics represent another frontier where polymer recycling intersects strongly with regulation. Waste electrical and electronic equipment (WEEE) is one of the fastest-growing waste streams globally, and plastics recovered from electronics often carry complex additive packages, including restricted substances such as legacy flame retardants [22]. This creates a situation where “technical recyclability” may be insufficient if recyclate does not comply with the chemical restrictions that govern new products and materials [23]. Bruno and Fiore [12] examined the recyclability of plastics from waste mobile phones in relation to EU regulatory frameworks (REACH and RoHS). The study placed material recovery in a compliance-oriented context, highlighting that polymer waste management strategies for WEEE must not only process yield or mechanical performance, but also integrate analytical screening, traceability, and decision rules for safe circulation.

End-of-life for plastics also includes biodegradation, particularly for single-use items and packaging. Yet the biodegradability of bioplastics is not a single intrinsic property, it depends on polymer chemistry, crystallinity, thickness, formulation, and the environmental conditions that control abiotic and biotic degradation pathways [24,25]. This complexity is the reason standardized methods (e.g., controlled biodegradation and disintegration tests and protocols) are widely used for certification. Gadaleta et al. [14] provided a comprehensive review of bioplastic biodegradability in managed and unmanaged environments, synthesizing evidence across settings and highlighting how test conditions and environmental variability shape observed outcomes. Complementing this broad perspective, Falzarano et al. [13] reported an experimental study on the anaerobic biodegradation of PLA-based disposable tableware under mesophilic conditions (38 °C) over 155 days, comparing biodegradation estimates derived from biogas production versus total organic carbon removal and reporting high biodegradation degrees when calculated by TOC removal (80.5–88.9%). Together, these two papers illustrate both the promise and the challenge of biodegradable alternatives, highlighting that meaningful biodegradation may occur under certain managed conditions and that robust choices should be made regarding material to provide a product that both fulfills technical requirements and is fully biodegradable.

In its entirety, this Special Issue reinforces a central message for future research: progress in plastic waste management will depend on coupling materials science with process engineering, analytics, and regulatory design, rather than treating these domains separately [26]. Several research directions appear particularly pressing. First, feedstock variability must be treated as a primary design constraint. Both mechanical and chemical recycling research should use realistic inputs and should report characterization in a way that supports comparability across studies [27,28]. Second, additive management deserves greater attention as a “circularity limiter”, not only in terms of performance stabilization but also in terms of migration, accumulation, and compliance in closed loops [29]. Third, the interface between recycling and biodegradation requires sharper boundaries and clearer communication. Biodegradability should be seen only for applications where its use makes sense and not as a “green” alternative that always works, especially in contexts where mismanagement and unclear communication can be overpowering. Finally, cross-cutting assessment tools (like life cycle assessment, traceability concepts, and harmonized standards) should be integrated earlier in research design so that laboratory advances translate into scalable, safe, and policy-aligned solutions [30].

Alongside the issues highlighted above, the role of Artificial Intelligence (AI) as a cross-cutting tool to support research in the field of polymeric materials is becoming increasingly important. Fang et al. [31] highlighted how machine/deep learning techniques are revolutionizing approaches to the characterization and selection of plastic waste. These techniques enable the automatic identification of polymers and additives using optical sensors and infrared spectroscopy with levels of accuracy that are difficult to achieve using conventional methods. This progress is a response to the high compositional variability of incoming plastic waste streams and the impacts this has on the quality of the recycled material as well as the reliability of recycling processes. AI is also beginning to play an important role in chemical recycling and thermochemical conversions (e.g., pyrolysis, gasification). Gabbar and Ahmad [32] highlighted how AI is already capable of increasing operational efficiency; it predicts yields and the composition of output products and optimizes operating parameters in the presence of heterogeneous feedstocks. This approach is particularly relevant for supply chains where the relationship between material structure, process conditions, and final properties of the product are non-linear and difficult to describe with simplified deterministic models—such as cross-linked polyurethanes or multi-source PET streams. Similarly, in the field of biological degradation and the design of biodegradable materials, AI is beginning to provide useful tools for correlating molecular structure, additives, environmental conditions and degradation kinetics, although this is still in the early stages [31]. However, the adoption of artificial intelligence also has structural limitations, such as the availability and quality of experimental data [33,34], difficulties in transferring models from the laboratory to industrial plants [35], the interpretability of results, and integration with regulatory constraints [36].

Circular management of polymeric materials depends not only on the development of new recycling technologies or new polymers, but also on the ability to integrate advanced digital tools into realistic experimental frameworks, comparable methodologies, and consistent industrial and regulatory strategies.
